# Extensive elastofibroma dorsi with scapulo-thoracic involvement in a male laborer: A case report

**DOI:** 10.1016/j.ijscr.2025.110883

**Published:** 2025-01-13

**Authors:** Faten Limaiem, Mohamed Amine Gharbi, Leila Bouhajja, Ramzi Bouzidi

**Affiliations:** a University of Tunis El Manar, Tunis Faculty of Medicine, 1007, Tunisia; b Pathology Department, Hospital Mongi Slim La Marsa, Tunisia; c Department of Orthopedic Surgery, Hospital Mongi Slim La Marsa, Tunisia; d Mohamed Kassab Institute of Orthopedics, Tunis, Tunisia

**Keywords:** Elastofibroma, Surgery, Pathology, Soft tissue mass, Benign tumor, case report

## Abstract

**Introduction and importance:**

Elastofibroma dorsi is a rare benign soft tissue lesion primarily located in the subscapular region. This distinctive lesion, with its unique radiological and histological features, poses diagnostic challenges due to its subtle presentation and overlap with other conditions.

**Case presentation:**

A 48-year-old man, manual laborer with an unremarkable medical history presented with a progressively enlarging mass below the right scapula over two years. Physical examination revealed a painless, soft, and mobile swelling near the scapular tip and along the chest wall. MRI demonstrated an elongated mass in the right posterolateral thoracic wall, characterized by smooth borders and a striped appearance on T1 and T2 sequences, consistent with elastofibroma. Surgical excision was performed, resulting in the removal of a 14 cm mass with a rubbery consistency and a gross appearance reminiscent of layered leaves. Histopathological analysis confirmed the diagnosis of elastofibroma.

**Clinical discussion:**

Elastofibroma, though usually asymptomatic, can present as a palpable mass in the scapular region. The characteristic striped appearance on MRI reflects alternating fibrous and fatty tissue. Histopathologically, elastofibroma is defined by abnormal elastic fibers within a fibrocollagenous matrix. Surgical excision is curative, and patients generally have an uneventful recovery with no further intervention required.

**Conclusions:**

This case emphasizes the need to recognize elastofibroma as a differential diagnosis for palpable masses near the scapula. Awareness of its clinical, imaging, and histopathological features is essential for accurate diagnosis and effective management, often involving surgical excision with favorable outcomes.

## Highlights

• Elastofibroma dorsi should be considered in patients with gradually enlarging masses in the infra- or periscapular region.
• MRI is the preferred diagnostic tool, revealing characteristic features such as well-defined masses with a striped appearance on T1 and T2 sequences.
• MRI findings help differentiate elastofibroma dorsi from other soft tissue tumors, including fibrolipoma and desmoid-type fibromatosis.
• The definitive diagnosis of elastofibroma dorsi is usually established through a post-surgical pathological examination.
• Regular observation is recommended for asymptomatic elastofibroma dorsi, while surgical excision is favored for symptomatic cases to ensure favorable outcomes.

## 1 Introduction

Elastofibroma dorsi (ED) is a rare benign soft tissue tumor commonly located in the periscapular region of the posterior chest wall [[1], [2], [3]]. Often asymptomatic, it is typically discovered incidentally on imaging, with larger lesions occasionally presenting as palpable chest wall masses. While the exact etiology of ED remains unclear, repetitive mechanical trauma in the subscapular area has been linked to its gradual development [4]. Magnetic Resonance Imaging (MRI) is the primary diagnostic tool for ED, with a definitive diagnosis often requiring post-surgical pathological examination. Significant knowledge gaps persist in understanding ED, particularly in its etiopathogenesis, diagnosis, and management, attributed to the rarity of cases and limited comprehensive studies. Moreover, diagnosing ED poses a challenge as it histologically mimics fibrolipoma and desmoid-type fibromatosis. Herein, we report a unique case of a large ED with scapulo-thoracic involvement in a 48-year-old male manual laborer. This report aims to elucidate ED's clinical presentation, diagnostic approaches, and therapeutic strategies to enhance clinical decision-making in similar cases.

This case report adheres to the SCARE Criteria [5].

## 2 Case presentation

### 2.1 Patient history and presenting complaint

A 48-year-old male manual laborer with an unremarkable past medical history, presented with a palpable mass beneath the right scapula that had gradually enlarged over the past two years.

### 2.2 Physical examination findings

Physical examination revealed a painless, soft, and movable swelling located near the tip of the scapula and the posterior chest wall.

### 2.3 Imaging findings

**Fig. 1:**
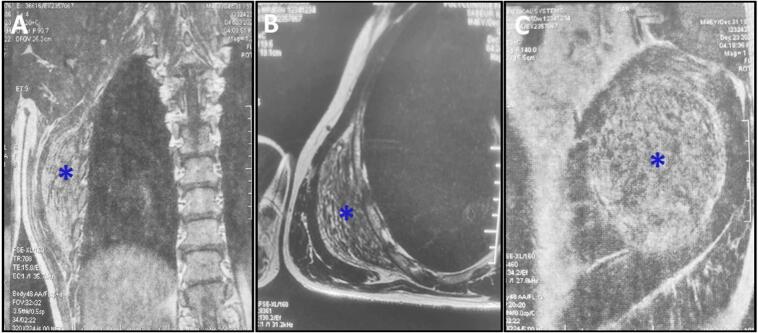
A,B, C: Magnetic resonance imaging depicting an elongated mass (blue asterisk) located in the right posterolateral thoracic wall, adjacent to the lower angle of the scapula. The mass (blue asterisk) displayed well-defined borders and a striated pattern, attributed to alternating linear structures with low signal intensity on T1 and T2 MRI sequences within a fatty background.

Magnetic resonance imaging showed an elongated mass in the right posterolateral thoracic wall, adjacent to the lower angle of the scapula. The mass displayed smooth borders and a striped appearance due to alternating linear structures with low signal intensity on both T1 and T2 MRI sequences, set against a fatty background (Fig. 1A, B, and C). These findings were consistent with a diagnosis of ED.

### 2.4 Surgery

**Fig. 2:**
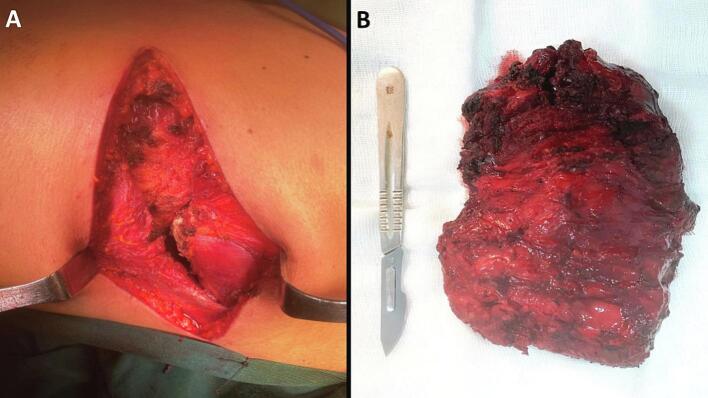
A: Intraoperative photos depict the tumor bed after resection, exposing the ribs and the scapulothoracic space following the separation of the scapula without caudal angulation. B: Macroscopic examination of the mass after complete excision, measuring 14 × 10 × 3 cm.

**Fig. 3:**
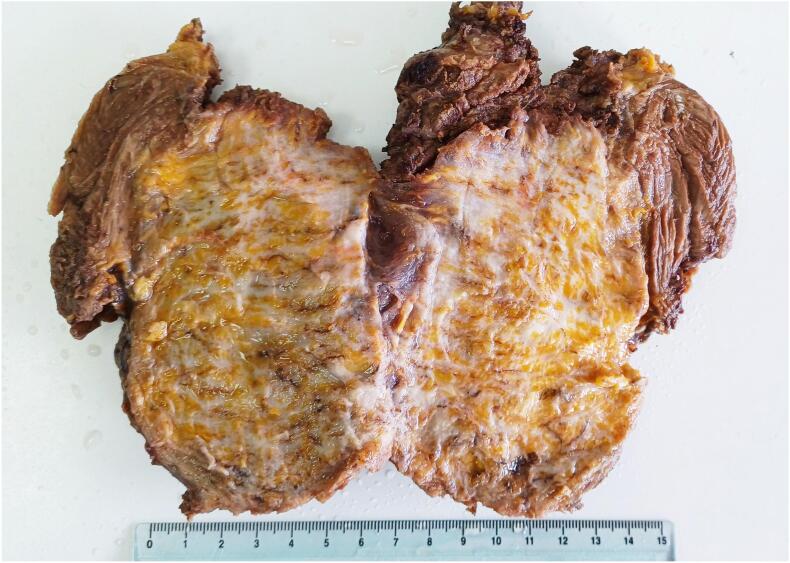
The cut surface displayed a gross appearance resembling layered leaves, with whitish fibrous tissue interspersed with streaks of yellow fatty tissue. (For interpretation of the references to colour in this figure legend, the reader is referred to the web version of this article.)

The patient subsequently underwent complete surgical excision of the mass (Fig. 2A). The excised specimen weighed 352 g, measured 14 × 10 × 3 cm, and exhibited a rubbery consistency (Fig. 2B). On the cut surface, the specimen displayed gray or whitish fibrous tissue interspersed with streaks of yellow fatty tissue, resembling layered leaves (Fig. 3).

### 2.5 Pathology findings

**Fig. 4:**
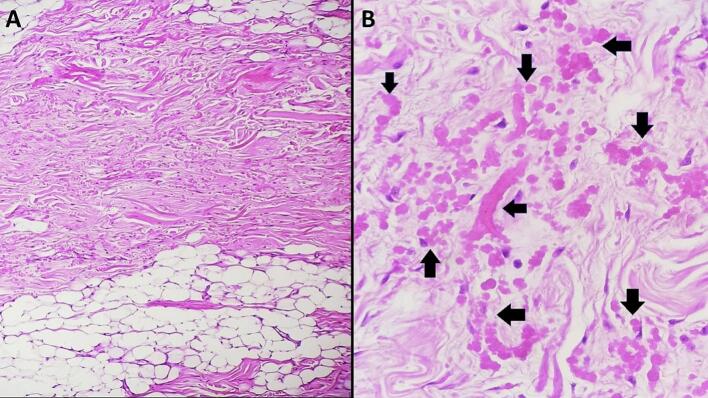
A: Histological examination of elastofibroma dorsi reveals a lesion characterized by the presence of fibrocollagenous tissue. Within this tissue, eosinophilic elastic fibers and scattered fibroblasts are observed. Additionally, a variable amount of mature adipose tissue is dispersed throughout the fibrocollagenous matrix**. (**Hematoxylin and eosin, magnification × 100**)**. B: Microscopic examination reveals elastic fibers with a coarse texture, exhibiting an eosinophilic (pink-stained) appearance and fragmented morphology (black arrows). These fibers have the propensity to align linearly, forming either globular or serrated disk-like structures that bear a resemblance to beads on a string (black arrows). **(**Hematoxylin and eosin, magnification × 400**)**. (For interpretation of the references to colour in this figure legend, the reader is referred to the web version of this article.)

Histopathological examination revealed an ill-defined proliferation of fibrocollagenous tissue containing numerous abnormal elastic fibers, along with scattered bland spindle cells mixed with adipose tissue (Fig. 4A). The elastic fibers were coarse, pink-stained, and fragmented, forming linearly arranged globular or serrated disk-like structures reminiscent of beads on a string (Fig. 4B). The final pathological diagnosis confirmed ED.

### 2.6 Postoperative course and follow-up

The patient had an uneventful postoperative recovery, and no further treatment was required. At the six-month follow-up, there was no evidence of mass recurrence. His surgical incision had healed properly and he was able to return to his normal activities and work without any restrictions.

## 3 Discussion

ED is classified within the fibroblastic/myofibroblastic tumor group in the 2020 World Health Organization Classification of Tumors of Soft Tissue and Bone [6]. Although ED is typically unilateral, bilateral cases occur in approximately 10 % of instances. In 99 % of cases, it is predominantly located between the scapula and the thoracic wall, nestled among the rhomboid major, latissimus dorsi, and serratus anterior muscles in the subscapular region [2]. Less frequently reported locations include the lateral chest wall, deltoid muscle, axilla, greater trochanter, olecranon, foot, tricuspid valve, tuberositas ischii, inguinal region, omentum majus, stomach, rectum, spinal canal, sclera, orbit, and mediastinum [3]. ED is most commonly diagnosed in older adults, typically between the ages of 50 and 70, with a notable predominance in females. Studies indicate that women are affected approximately three times more frequently than men, resulting in a female-to-male ratio of about 3:1. The classification of ED as a true tumor remains a subject of debate. While the exact pathogenesis of ED is unclear, it is traditionally thought to result from pseudotumor formation due to repetitive mechanical stress at the junction of the scapula and thoracic wall. This stress can induce microtrauma, leading to excessive elastic tissue production by fibroblasts [7,8]. Consequently, research has investigated the potential link between manual labor or strenuous activities and the onset of ED, as noted in our patient. However, the occurrence of ED in atypical locations such as the mediastinum and omentum, challenges this notion. Some researchers believe that genetic variations, including mutations in regions like Xq12-q22 and chromosome 19, may also contribute to the development of ED [7]. ED often evolves asymptomatically, with gradual growth observed in over half of the cases. When symptoms do manifest, they may include shoulder swelling, increased visibility during shoulder movement, scapular displacement, and pain. Ultrasonography is the preferred initial imaging method for symptomatic patients due to its non-invasive nature and accessibility. On ultrasound, ED typically appears echogenic, exhibiting features such as linear hypoechoic areas of fat within fibroelastic tissue or presenting as a homogeneous hypoechoic mass [9]. In practice, over 50 % of patients are asymptomatic and are incidentally detected on CT or MRI scans [10]. Computed tomography (CT) reveals ED as an inhomogeneous soft tissue mass in the subscapular region, characterized by lower-density linear fatty tissue structures. On T1- and T2-weighted MRI, ED closely resembles muscle tissue with linear fatty signal changes and shows heterogeneous contrast enhancement with gadolinium [11]. Benign characteristics of the mass can be identified through diffusion-weighted imaging [11]. The utility of preoperative biopsy for ED is a topic of debate. Hayes et al. [12] advocate for core biopsy to obtain a tissue diagnosis prior to treatment, while Massengill et al. [13] suggest that clinical and radiological findings alone may be sufficient for an accurate diagnosis. The management of ED predominantly entails surgical intervention, especially for symptomatic cases or those surpassing 5 cm in size. Surgical excision, employing marginal resection as the preferred technique, stands as the conventional method known for yielding positive results, characterized by low complication and recurrence rates [14]. If ED remains asymptomatic, observation may be appropriate. Regular monitoring is essential to detect any changes in size or symptoms. Some authors contend that surgical excision is necessary for pathological verification, regardless of symptoms [14]. Pathological confirmation is typically achieved through examination of the excised specimen. ED exhibits distinctive macroscopic and histological features. Macroscopically, it typically presents as a poorly defined, firm to rubbery mass with gray-white fibrous tissue interspersed with pockets of yellow adipose tissue upon sectioning [6]. Histologically, ED is characterized by its unique composition, primarily consisting of elastic fibers and collagen. The tumor usually demonstrates low to moderate cellularity, featuring spindle-shaped fibroblasts within a dense fibrous stroma marked by thick eosinophilic collagen bundles that contribute to the tumor's overall structure. Mature adipocytes are often present within the tumor matrix. A hallmark of ED is the abundant mature elastic fibers, which appear as densely eosinophilic globules and elongated structures with irregular, fuzzy outlines or beaded cords. These elastic fibers can be effectively visualized using special stains, such as Verhoeff-Van Gieson. Immunohistochemical analysis reveals that spindle cells are positive for vimentin, CD34, and lysozyme, while markers such as smooth muscle actin, desmin, and S-100 protein are not expressed [15]. The differential diagnosis of ED includes fibrolipoma, a variant of lipoma characterized by a significant fibrous tissue component alongside mature adipose tissue, and desmoid-type fibromatosis, a locally aggressive fibroblastic neoplasm that can arise in the subscapular region [15]. Spontaneous regression of ED is exceedingly rare, with no documented cases of metastasis or malignant transformation. Recurrence rates in the literature range from 0.06 % to 4.5 % [16]. The most commonly reported complications following ED resection include hematoma and seroma formation, occurring in 40 % to 50 % of cases, with a direct correlation to tumor size [16]. Although there is no consensus on postoperative rehabilitation, immobilization of the area for one week is generally recommended.

## 4 Conclusion

In summary, this case report emphasizes the need to recognize ED as a potential diagnosis in patients presenting with progressively enlarging masses in the infra- or periscapular region. Our findings illustrate that MRI is an essential diagnostic tool, showcasing unique imaging features that help differentiate ED from other soft tissue tumors. Diagnosis is confirmed through post-surgical pathological examination, with surgical excision being the preferred treatment for symptomatic cases. This case highlights the need for clinicians to maintain a high index of suspicion for ED, as early recognition and appropriate management can lead to favorable outcomes and resolution of symptoms. Ongoing awareness of its clinical presentation, imaging characteristics, and histopathological findings is crucial for optimal patient care.

## Author contribution

**Dr. Faten LIMAIEM** and **Dr Leila BOUHAJJA:** Prepared, organized, wrote, and edited all aspects of the manuscript.

**Dr. Mohamed Amine GHARBI,** and **Pr. Ramzi BOUZIDI:** Read, edited, and approved the final version of the manuscript. Contributed to data acquisition, analysis, and interpretation. Provided final approval of the manuscript before its submission.

## Consent statement

Written informed consent was obtained from the patient for publication of this case report and accompanying images. A copy of the written consent is available for review by the Editor-in-Chief of this journal on request.

## Ethical approval

Ethical approval for this study was provided by the Ethical Committee of Mongi Slim University Hospital, Marsa, Tunisia.

## Guarantor

Dr. Faten LIMAIEM.

## Provenance and peer review

Not commissioned, externally peer-reviewed.

## Funding

This research did not receive any specific grant from funding agencies in the public, commercial, or not-for-profit sectors.

## Declaration of competing interest

None declared.
